# Characteristics and risk factors of glycemic control among middle-aged and elderly diabetes patients under management: a cross-sectional survey based on national essential public health services in Mianyang City

**DOI:** 10.1186/s12902-026-02465-x

**Published:** 2026-08-12

**Authors:** Wenjuan Xiao, Fei Tong, Peiyang Mao, Bingjie Qi, Tianyu Yang, Zhuo Wang, Yinghong Shi

**Affiliations:** 1 Mianyang Center for Disease Control and Prevention, Mianyang, Sichuan P. R. China; 2 Sichuan Center for Disease Control and Prevention, Chengdu, Sichuan P. R. China

**Keywords:** Type 2 diabetes mellitus, Demographic characteristics, Glycemic control, Risk factors

## Abstract

**Background:**

This study aimed to explore the demographic characteristics and risk factors of glycemic control among type 2 diabetes mellitus (T2DM) patients aged 40 years and above enrolled in the National Essential Public Health Services (NEPHS) in China, providing scientific evidence for further improving grassroots glycemic control management.

**Methods:**

A stratified multi-stage sampling method was used to select 1500 patients. Their diabetes management files were retrieved, and questionnaire surveys, physical examinations, and laboratory tests were conducted simultaneously. Skewed quantitative variables were described as median (25th percentile, 75th percentile), and nonparametric rank-sum tests were used for group comparisons. Categorical variables were expressed as frequencies and percentages (%) with group comparisons using χ² tests. Logistic regression with Huber–White (sandwich) standard errors was performed to identify risk factors for glycemic control. Furthermore, interaction models (knowledge level × insulin injection, blood glucose monitoring adherence× insulin injection) were established to evaluate the stratified effects.

**Results:**

49.5% of patients achieved good glycemic control (HbA1c < 7.0%). Multivariate analysis revealed that age ≥ 60 years (OR = 1.41, 95% CI: 1.12–1.77, *P* = 0.003), rural (OR = 1.33, 95% CI: 1.06–1.66, *P* = 0.012), regular physical exercise (OR = 1.51, 95% CI: 1.21–1.88, *P* < 0.001), periodic HbA1c monitoring (1 time/year: OR = 1.44, 95% CI: 1.11–1.89, *P* = 0.007; ≥4 times/year: OR = 1.93, 95% CI: 1.31–2.85, *P* = 0.001), and comorbid hypertension (OR = 1.31, 95% CI: 1.06–1.62, *P* = 0.015) were independently cross-sectionally associated with good glycemic control. Conversely, diabetes duration ≥ 10 years (OR = 0.67, 95% CI: 0.50–0.89, *P* = 0.006), current smoking (OR = 0.66, 95% CI: 0.48–0.91, *P* = 0.010), moderate knowledge level (OR = 0.61, 95% CI: 0.46–0.81, *P* = 0.001), treatment status (oral antidiabetic agents only: OR = 0.49, 95% CI: 0.24–0.98, *P* = 0.045; insulin only: OR = 0.36, 95% CI: 0.15–0.86, *P* = 0.022), and high blood glucose monitoring adherence (OR = 0.59, 95% CI: 0.41–0.86, *P* = 0.006) were cross-sectionally associated with poor glycemic control. Interaction analysis demonstrated a significant positive interaction between high knowledge level and insulin therapy (OR = 4.60, 95% CI: 1.78–11.89, *P* = 0.002). No significant interaction was observed between blood glucose monitoring adherence and insulin therapy (*P* > 0.05).

**Conclusion:**

Glycemic control among aged ≥ 40 years with T2DM under the NEPHS remains suboptimal. Strengthened proactive clinical monitoring and integrated multimorbidity care are warranted to improve primary care diabetes outcomes. The observed knowledge–treatment interaction, while suggesting potential for precision stratified management, requires confirmation through prospective or interventional studies before implementation.

**Supplementary Information:**

The online version contains supplementary material available at https://doi.org/10.1186/s12902-026-02465-x.

## Introduction

Type 2 diabetes mellitus (T2DM) has emerged as one of the most formidable public health challenges of the twenty-first century. China, home to the world’s largest diabetes population, continues to experience a mounting disease burden. In 2023, an estimated 233 million individuals in China were living with diabetes, more than double the 88.48 million in 2005—a 163% increase in absolute numbers [1]. Population aging serves as the primary driver of this epidemic: the 2018 national survey reported diabetes prevalence of 23.9% among adults aged 60–69 years, 27.3% among those aged 70 years and above, and over 35% among individuals aged 80 years and older [2]. Yet, the epidemiological trajectory among middle-aged adults warrants equal attention. The prevalence among those aged 40–49 years has surged to 11.1%, nearly doubling from 6.5% observed in the 30–39 years age group [2], signaling entry into an accelerated phase of disease burden accumulation. From the perspective of disease natural history, T2DM typically presents a 10- to 15-year window of opportunity between diagnosis and the onset of clinically apparent complications. The UK Prospective Diabetes Study (UKPDS) and its 24-year post-trial follow-up established that early intensive glycemic control produces a durable “legacy effect” or “metabolic memory,” with sustained benefits including a 24% reduction in microvascular complications and a 15% reduction in myocardial infarction risk persisting for more than a decade [3]. For patients aged 40–59 years, this critical window coincides precisely with their transition into older age. Achieving optimal glycemic control during this phase could maximally delay complication onset, thereby reducing future disease burden and care dependency in later life. Consequently, adults aged 40 years and above represent both an early warning population for accelerated diabetes burden accumulation and a strategic target for intervention to promote healthy aging.

Although T2DM remains incurable, standardized management can achieve satisfactory glycemic control and delay complication progression. However, the current landscape of glycemic control in China is characterized by overall stagnation coupled with substantial regional heterogeneity. The 2018–2019 national survey revealed that merely 50.1% of treated patients achieved glycemic targets, showing virtually no improvement compared with 2013 [2]. Marked regional disparities persist: a 2016 rural survey in Zhejiang Province, an economically developed eastern region, reported a control rate of 47.89% [4], whereas a 2021 rural survey in Guangxi, a less developed western region, found only 22.68% [5]. Notably, with the near-universal coverage of basic public health services, such disparities may reflect less on healthcare accessibility than on the quality and effectiveness of routine management—specifically, how institutional resources are translated into tangible health outcomes at the primary care level.

In China, the National Essential Public Health Services (NEPHS) program provides an institutionalized care pathway for T2DM patients. Enrolled individuals are entitled to four free fasting plasma glucose tests annually, at least four face-to-face follow-up visits per year, and targeted health education delivered by primary healthcare institutions. Notably, glycated hemoglobin (HbA1c)—the gold standard for glycemic assessment per the Chinese Diabetes Society guidelines—is not mandated as a routine NEPHS output. This policy-practice gap in glycemic monitoring renders the NEPHS a valuable natural experiment for examining how real-world access to different glycemic monitoring shapes glycemic control outcomes. Previous studies have identified inadequate patient knowledge, poor self-management compliance, and unhealthy lifestyles as core barriers to glycemic control [6–9]. However, these investigations were predominantly conducted in hospital settings or among general populations without age stratification. In-depth analysis targeting adults aged 40 years and above within this real-world management context remains scarce.

This study focuses on T2DM patients aged 40 years and older enrolled in the NEPHS framework. We systematically characterize their demographic profiles, disease cognition, self-management behaviors, and lifestyle patterns, and explore key determinants of glycemic control using robust logistic regression and interaction modeling. Drawing upon the Health Belief Model and self-efficacy theory, we specifically examine the potential synergistic effect between diabetes knowledge and treatment status. By identifying actionable, stratifiable factors, this study aims to generate evidence-based recommendations for optimizing precision management strategies within the NEPHS framework and enhancing the effectiveness of primary care diabetes prevention and control.

## Materials and methods

### Study design

The data for this study were derived from the Mianyang City National Essential Public Health Service (NEPHS) Program Effectiveness Evaluation. Led by the Mianyang Municipal Health Commission and implemented by the Mianyang Center for Disease Control and Prevention, this evaluation was conducted from December 2024 to March 2025 across the entire city to systematically assess the quality of diabetes management within the NEPHS framework. This study utilized de-identified secondary data collected during this evaluation to analyze the current status of glycemic control and its influencing factors among T2DM patients aged ≥ 40 years under standardized management.

### Study setting

Mianyang City is located in northwestern Sichuan Province, with a permanent resident population of approximately 4.9 million. The city comprises 9 districts/counties with varying population densities, providing a representative setting for examining urban-rural disparities in diabetes management. The NEPHS program has been fully implemented across all township-level health facilities in Mianyang since 2009.

### Participants

The study population comprised T2DM patients aged ≥ 40 years who were under standardized management of the NEPHS program in Mianyang City.

Inclusion criteria were: (a) confirmed diagnosis of T2DM by secondary or higher-level medical institutions, in accordance with the then-current diagnostic criteria in China; (b) permanent residents of Mianyang City (cumulative residence ≥ 6 months in the past 12 months); (c) age ≥ 40 years at the time of survey; and (d) provision of written informed consent.

Exclusion criteria were: (a) residence in functional communities (military barracks, student dormitories, nursing homes, construction sheds) where household follow-up was infeasible; (b) severe cognitive impairment or psychiatric disorders precluding independent completion of questionnaires; (c) pregnancy or lactation; and (d) acute diabetic complications (ketoacidosis, hyperosmolar hyperglycemic state) or severe infection within the preceding 3 months.

### Sampling method

The sample size was administratively determined at 1,500 participants (30 sites × 50 participants) based on field logistics, budget, and the capacity of the evaluation team. This sample size exceeds the theoretical minimum of approximately 854 participants required for estimating a proportion of 50.1% with a 95% confidence interval width of ± 5% points, assuming simple random sampling and accounting for a design effect of 2.0 and 10% non-response [10]. However, because the second stage employed non-probability sampling, this calculation serves only as a rough benchmark rather than a guarantee of statistical precision. The findings should be interpreted as characterizing an available sample of actively managed patients rather than as population parameter estimates.

### Measures

Data were obtained from two sources: (a) NEPHS management records, extracting date of diagnosis (to calculate disease duration) and physician-rated medication adherence (regular, intermittent, or non-adherent); and (b) a specialized survey conducted by uniformly trained investigators using questionnaires(Supplementary File 1) and operational protocols, comprising three components: questionnaire survey, physical examination, and laboratory testing.

Questionnaire survey collected sociodemographic characteristics (age, sex, education level, occupation, household per capita monthly income), disease-related information (family history, self-reported complications), self-management behaviors (blood glucose monitoring frequency, medication behaviors), and lifestyle factors (dietary patterns, exercise habits, smoking, alcohol consumption).

Diabetes-related knowledge was assessed using a self-developed questionnaire consisting of 16 items across four dimensions: (a) basic knowledge (4 items), (b) symptom recognition (2 items), (c) complication awareness (1 item), and (d) self-management knowledge (9 items). Scoring rules: Single-choice items scored 1 for correct answers and 0 for incorrect or “don’t know” responses; multiple-choice items were scored proportionally based on the ratio of correctly selected options to total correct options. The summed item scores were converted to a percentage scale (0–100), with higher scores indicating better knowledge mastery. Participants were classified into three groups: low (< 60 points), moderate (60–79 points), and high (≥ 80 points) knowledge levels.

Physical examination followed standardized protocols for height (precision 0.1 cm), weight (precision 0.1 kg), waist circumference (precision 0.1 cm), and blood pressure. Blood pressure was measured using a calibrated electronic sphygmomanometer, with three readings taken at 1-minute intervals and the mean value recorded.Laboratory testing: Fasting venous blood samples (≥ 8 h fasting) were collected and transported to a qualified laboratory for measurement of fasting blood glucose and glycated hemoglobin (HbA1c).

### Data analysis

Data analysis was conducted using STATA 18.0. All continuous variables deviated from normal distribution (Shapiro-Wilk test, W = 0.677–0.998, all *P* < 0.05) and were described as median (25th percentile, 75th percentile) [M (P25, P75)]. Between-group comparisons were performed using Mann-Whitney U test (two groups) or Kruskal-Wallis H test (multiple groups). Categorical variables were expressed as frequencies (proportions %) and compared using Pearson χ² test or Fisher’s exact test (when > 20% of cells had expected frequencies < 5).

Multivariate analysis employed a robust binary logistic regression model (Huber-White sandwich estimator), with good glycemic control (HbA1c < 7.0%) as the dependent variable (1 = good, 0 = poor).

Interaction analysis examined effect modification through two independent models:Model 1:Knowledge level (three categories, low as reference), insulin therapy (binary, no as reference), and their product term (knowledge level × insulin therapy);Model 2: Blood glucose monitoring adherence (three categories, low adherence as reference), insulin therapy, and their product term (adherence × insulin therapy).

Both models were adjusted for age, sex, disease duration, current smoking, regular exercise, and comorbid hypertension as potential confounders. Interaction effects were evaluated by the OR and 95% CI of the product term, with statistical significance set at *P* < 0.05. The significance level for all tests was α = 0.05 (two-tailed).

### Ethical considerations

This study used de-identified secondary data originally collected by the Mianyang Municipal Health Commission as part of routine public health service evaluation. For the secondary analysis presented in this manuscript, ethical approval was obtained from the Ethics Committee of the Mianyang Center for Disease Control and Prevention (approval number: MYCDC202602).

## Results

### General characteristics

A total of 1,500 patients with type 2 diabetes mellitus enrolled in the NEPHS were included in this study. Of these, 742 (49.47%) were classified into the good glycemic control group and 758 (50.53%) into the poor glycemic control group. Comparisons between the two groups regarding demographic characteristics, clinical features, behaviors and knowledge, and healthcare practices are presented in Table 1.

### Demographic characteristics

Patients aged 60 years and older accounted for 60.93% (914/1,500) of the total sample. The proportion of patients aged ≥ 60 years was higher in the good control group (65.09%) than in the poor control group (56.86%), and this difference was statistically significant (χ² = 10.680, *P* = 0.001). With respect to urban-rural distribution, rural residents comprised 56.67% (850/1,500) of the total cohort; the proportion of rural patients was higher in the good control group (59.30%) than in the poor control group (54.09%) (χ² = 4.144, *P* = 0.042). No statistically significant differences were observed between the two groups in terms of sex, education level, occupation, annual household income, or number of cohabitants (*P* > 0.05).

### Clinical characteristics 

The median duration of diabetes was 7 years (interquartile range [IQR], 4–10) in the good control group and 8 years (IQR, 5–11) in the poor control group, with a statistically significant difference between groups (Z = 2.726, *P* = 0.006). The proportion of patients with comorbid hypertension was higher in the good control group (59.16%) than in the poor control group (50.79%) (χ² = 10.618, *P* = 0.001). No statistically significant differences were found between groups for family history of diabetes, BMI category, abdominal obesity, systolic blood pressure, diastolic blood pressure, or diabetic complications (*P* > 0.05).

### Behaviors and knowledge

The proportion of current smokers was lower in the good control group (13.48%) than in the poor control group (18.92%) (χ² = 8.149, *P* = 0.004). Current alcohol consumption was also less prevalent in the good control group (23.99% vs. 28.93%, χ² = 4.698, *P* = 0.030), whereas regular physical exercise was more common (63.87% vs. 57.80%, χ² = 5.769, *P* = 0.016). No statistically significant difference was observed between groups in adherence to a low-salt, low-fat diet (*P* = 0.948). The total diabetes knowledge score was significantly lower in the good control group (median, 44.64; IQR, 27.81–59.58) compared with the poor control group (median, 48.23; IQR, 32.08–63.96) (Z = 3.104, *P* = 0.002). Specifically, scores for basic knowledge, symptom-related knowledge, and management knowledge were all higher in the poor control group than in the good control group (all *P* < 0.05), whereas no significant difference was observed in complication-related knowledge scores (*P* = 0.088).

### Healthcare practices

Medication adherence (physician-assessed) differed significantly between the two groups (χ² = 7.853, *P* = 0.020), with 84.24% of all patients reporting regular medication use. Regarding HbA1c monitoring frequency, the proportion of patients who underwent ≥ 4 measurements per year was higher in the good control group (11.34%) than in the poor control group (6.99%) (χ² = 12.115, *P* = 0.007). Treatment status also differed significantly between groups (χ² = 9.837, *P* = 0.020); the good control group had slightly higher proportions of patients receiving no pharmacological treatment. The proportion of patients who self-rated their blood glucose monitoring adherence as “good” was lower in the good control group (64.46%) than in the poor control group (70.24%) (χ² = 5.681, *P* = 0.017).

**Table 1:** Comparison of basic characteristics of diabetic patients by glycemic control status

Characteristic	Overall (*N* = 1500)	Good control group (*N* = 742)	Poor control group (*N* = 758)	z/χ² value	*P* value
**Demographic characteristics**					
Age group (years)				10.680	**0.001**
< 60	586 (39.07)	259 (34.91)	327 (43.14)		
≥ 60	914 (60.93)	483 (65.09)	431 (56.86)		
Sex				0.170	0.680
Male	750 (50.00)	375 (50.54)	375 (49.47)		
Female	750 (50.00)	367 (49.46)	383 (50.53)		
Urban-rural residence				4.144	**0.042**
Urban	650 (43.33)	302 (40.70)	348 (45.91)		
Rural	850 (56.67)	440 (59.30)	410 (54.09)		
Education level				1.351	0.245
Primary school and below	990 (66.18)	501 (67.61)	489 (64.77)		
Junior high school and above	506 (33.82)	240 (32.39)	266 (35.23)		
Occupation				0.071	0.789
Farmer	1016 (67.73)	505 (68.06)	511 (67.41)		
Other	484 (32.27)	237 (31.94)	247 (32.59)		
Annual household income (yuan)				0.975	0.323
< 50,000	1235 (82.44)	619 (83.42)	616 (81.48)		
≥ 50,000	263 (17.56)	123 (16.58)	140 (18.52)		
Number of cohabitants	2 (2, 4)	2 (2, 4)	2 (2, 4)	0.790	0.429
**Clinical characteristics**					
Duration of diabetes (years)	7 (4, 11)	7 (4, 10)	8 (5, 11)	2.726	**0.006**
Family history of diabetes				2.033	0.154
No	968 (64.71)	492 (66.49)	476 (62.96)		
Yes	528 (35.29)	248 (33.51)	280 (37.04)		
BMI category				1.489	0.685
Underweight	25 (1.66)	11 (1.48)	14 (1.85)		
Normal	490 (32.67)	233 (31.40)	257 (33.91)		
Overweight	663 (44.20)	335 (45.15)	328 (43.26)		
Obese	322 (21.47)	163 (21.97)	159 (20.98)		
Abdominal obesity				2.028	0.154
No	794 (52.93)	379 (51.08)	415 (54.75)		
Yes	706 (47.07)	363 (48.92)	343 (45.25)		
Systolic blood pressure (mmHg)	141 (130, 154)	140 (129, 153)	142 (131, 154)	1.306	0.192
Diastolic blood pressure (mmHg)	81 (75, 89)	81 (75, 88)	82 (75, 89)	0.843	0.400
Diabetic complications (foot, nephropathy, retinopathy, and neuropathy)				0.000	0.984
No	1387 (92.47)	686 (92.45)	701 (92.48)		
Yes	113 (7.53)	56 (7.55)	57 (7.52)		
Comorbid hypertension				10.618	**0.001**
No	676 (45.07)	303 (40.84)	373 (49.21)		
Yes	824 (54.93)	439 (59.16)	385 (50.79)		
**Behaviors and knowledge**					
Current smoking				8.149	**0.004**
No	1255 (83.78)	642 (86.52)	613 (81.08)		
Yes	243 (16.22)	100 (13.48)	143 (18.92)		
Current alcohol consumption				4.698	**0.030**
No	1102 (73.52)	564 (76.01)	538 (71.07)		
Yes	397 (26.48)	178 (23.99)	219 (28.93)		
Regular physical exercise				5.769	**0.016**
No	586 (39.20)	267 (36.13)	319 (42.20)		
Yes	909 (60.80)	472 (63.87)	437 (57.80)		
Low-salt, low-fat diet				0.004	0.948
No	367 (24.47)	181 (24.39)	186 (24.54)		
Yes	1133 (75.53)	561 (75.61)	572 (75.46)		
Diabetes knowledge score					
Total score	46.09 (30.16, 61.88)	44.64 (27.81, 59.58)	48.23 (32.08, 63.96)	3.104	**0.002**
Basic knowledge	50.00 (25.00, 75.00)	50.00 (25.00, 75.00)	50.00 (25.00, 75.00)	3.395	**< 0.001**
Symptom knowledge	36.67 (13.33, 55.00)	34.17 (10.00, 55.00)	36.67 (16.67, 55.00)	2.299	**0.022**
Complication knowledge	20.00 (0.00, 40.00)	10.00 (0.00, 40.00)	20.00 (0.00, 40.00)	1.706	0.088
Management knowledge	51.48 (31.11, 67.04)	50.37 (26.67, 66.11)	52.78 (33.52, 67.96)	1.974	**0.048**
**Healthcare practices**					
Medication adherence (physician-assessed)				7.853	**0.020**
Regular	1261 (84.24)	622 (83.94)	639 (84.52)		
Intermittent	155 (10.35)	68 (9.18)	87 (11.51)		
Non-adherent	81 (5.41)	51 (6.88)	30 (3.97)		
HbA1c monitoring				12.115	**0.007**
Not measured	918 (61.24)	427 (57.62)	491 (64.78)		
Once/year	353 (23.55)	185 (24.97)	168 (22.16)		
Twice/year	91 (6.07)	45 (6.07)	46 (6.07)		
≥ 4 times/year	137 (9.14)	84 (11.34)	53 (6.99)		
Treatment status				9.837	**0.020**
No medication	51 (3.40)	34 (4.58)	17 (2.24)		
Oral agents only	409 (27.27)	196 (26.42)	213 (28.10)		
Insulin only	54 (3.60)	20 (2.70)	34 (4.49)		
Combination therapy	986 (65.73)	492 (66.31)	494 (65.17)		
Blood glucose monitoring adherence (self-reported)				5.681	**0.017**
Poor	488 (32.62)	263 (35.54)	225 (29.76)		
Good	1008 (67.38)	477 (64.46)	531 (70.24)		

Note: All continuous variables deviated from normal distribution by the Shapiro-Wilk test (W = 0.872–0.998, all *P* < 0.05) and were described as median (interquartile range), with between-group comparisons performed using non-parametric tests (Mann-Whitney U, Kruskal-Wallis H). Percentages for categorical variables were calculated based on the number of valid (non-missing) observations for each characteristic. Missing data ranged from 0.07% to 0.33% across variables. And were presented as frequency (percentage) with between-group comparisons performed using the Pearson χ² test. Bold values indicate *P* < 0.05 BMI, Body Mass Index. Underweight (BMI<18.5), Normal (18.5≤BMI<24), Overweight (24≤BMI<28), and Obese (BMI≥28). Criteria for abdominal obesity: male waist circumference ≥90 cm; female waist circumference ≥85 cm. Diabetes knowledge score was converted to a percentage-based total score (0–100) by summing scores from all items across each domain, with higher scores indicating better knowledge. Combination therapy in the treatment status refers to patients who received two or more of the following measures: oral hypoglycemic agents, insulin injection, and lifestyle intervention

### Factors associated with glycemic control

Glycemic control status was used as the dependent variable (1 = good control, 0 = poor control). Variables with *P* < 0.05 in the univariate analysis and those deemed clinically important were entered into a multivariable logistic regression model. Variable assignments are shown in Table 2, and the regression results are presented in Table 3.

The results indicated that age ≥ 60 years (OR = 1.41, 95% CI: 1.12–1.77, *P* = 0.003), rural residence (OR = 1.33, 95% CI: 1.06–1.66, *P* = 0.012), regular physical exercise (OR = 1.51, 95% CI: 1.21–1.88, *P* < 0.001), HbA1c monitoring once per year (OR = 1.44, 95% CI: 1.11–1.89, *P* = 0.007) and ≥ 4 times per year (OR = 1.93, 95% CI: 1.31–2.85, *P* = 0.001), and comorbid hypertension (OR = 1.31, 95% CI: 1.06–1.62, *P* = 0.015) are associated with higher odds of good glycemic control.

Conversely, disease duration ≥ 10 years (OR = 0.67, 95% CI: 0.50–0.89, *P* = 0.006), current smoking (OR = 0.66, 95% CI: 0.48–0.91, *P* = 0.010), treatment with oral hypoglycemic agents only (OR = 0.49, 95% CI: 0.24–0.98, *P* = 0.045), insulin therapy only (OR = 0.36, 95% CI: 0.15–0.86, *P* = 0.022), and self-rated “high” blood glucose monitoring adherence (OR = 0.59, 95% CI: 0.41–0.86, *P* = 0.006) are associated with lower odds of good glycemic control. Sex, current alcohol consumption, low-salt low-fat diet, knowledge level (medium and high), combination therapy (marginally significant), medication adherence (intermittent use and non-adherence), HbA1c monitoring twice per year, and “moderate” blood glucose monitoring adherence showed no statistically significant association with glycemic control (*P* > 0.05).

**Table 2:** Variable assignment for the logistic regression model

Variable	Assignment
Glycemic control status	0 = Poor control, 1 = Good control
Age group (years)	1 = < 60, 2 = ≥ 60
Sex	1 = Male, 2 = Female
Urban-rural residence	1 = Urban, 2 = Rural
Disease duration (years)	1 = < 5, 2 = 5–10, 3 = ≥ 10 years
Current Smoking	0 = Current non-smoker, 1 = Current smoker
Alcohol consumption	0 = Current non-drinker, 1 = Occasional or frequent drinker
Regular physical exercise	0 = Irregular/No exercise, 1 = Regular exercise
Low-salt, low-fat diet	0 = No, 1 = Yes
Treatment status	0 = No medication, 1 = Oral agents only, 2 = Insulin only, 3 = Combination therapy
Blood glucose monitoring adherence	1 = Low, 2 = Medium, 3 = High
Comorbid hypertension	0 = No, 1 = Yes
HbA1c monitoring	1 = Not measured, 2 = Once/year, 3 = Twice/year, 4 = ≥ 4 times/year
Medication adherence (physician-assessed)	1 = Regular, 2 = Intermittent, 3 = Non-adherent
Knowledge level	1 = Low, 2 = Medium, 3 = High

**Table 3:** Multivariable logistic regression analysis of factors associated with glycemic control** (***N* = 1490**)**

Variable	OR	SE	z value	*P* value	95% CI	
					Lower	Upper
Age group (years) (Reference: <60)						
≥ 60	1.41	0.16	2.93	**0.003**	1.12	1.77
Sex (Reference: Male)						
Female	0.81	0.10	-1.73	0.084	0.63	1.03
Urban-rural residence (Reference: Urban)						
Rural	1.33	0.15	2.51	**0.012**	1.06	1.66
Duration of diabetes (Reference: <5 years)						
5–10 years	0.86	0.12	-1.12	0.263	0.65	1.12
≥ 10 years	0.67	0.10	-2.74	**0.006**	0.50	0.89
Current smoking (Reference: No)						
Yes	0.66	0.11	-2.57	**0.010**	0.48	0.91
Current alcohol consumption (Reference: No)						
Yes	0.82	0.11	-1.44	0.151	0.62	1.08
Regular physical exercise (Reference: No)						
Yes	1.51	0.17	3.60	**< 0.001**	1.21	1.88
Low-salt, low-fat diet (Reference: No)						
Yes	0.92	0.12	-0.68	0.496	0.71	1.18
Knowledge level (Reference: Low)						
Medium	0.61	0.09	-3.42	**0.001**	0.46	0.81
High	0.86	0.20	-0.61	0.539	0.54	1.38
Treatment status (Reference: No medication)						
Oral agents only	0.49	0.17	-2.01	**0.045**	0.24	0.98
Insulin only	0.36	0.16	-2.29	**0.022**	0.15	0.86
Combination therapy	0.50	0.18	-1.94	0.052	0.25	1.01
Medication adherence (physician-assessed) (Reference: Regular)						
Intermittent	0.76	0.14	-1.48	0.139	0.54	1.09
Non-adherent	1.24	0.36	0.74	0.459	0.70	2.20
HbA1c monitoring (Reference: Not measured)						
Once/year	1.44	0.20	2.69	**0.007**	1.11	1.89
Twice/year	1.33	0.32	1.19	0.233	0.83	2.12
≥ 4 times/year	1.93	0.38	3.30	**0.001**	1.31	2.85
Blood glucose monitoring adherence (Reference: Low)						
Medium	0.76	0.16	-1.33	0.183	0.50	1.14
High	0.59	0.11	-2.75	**0.006**	0.41	0.86
Comorbid hypertension (Reference: No)						
Yes	1.31	0.14	2.44	**0.015**	1.06	1.62

Note: The dependent variable was glycemic control status (1 = Good control, 0 = Poor control). OR, odds ratio; 95% CI, 95% confidence interval. The model was adjusted for age, sex, urban-rural residence, duration of diabetes, current smoking, regular physical exercise, knowledge level, treatment status, blood glucose monitoring adherence, hypertension, and other variables. Robust standard errors were used. Knowledge level was categorized into low (< 60 points), medium (60–79 points), and high (≥ 80 points) based on the percentage-based total score. Regular physical exercise was defined as exercise ≥ 3 times/week and ≥ 30 min/session. Bold values indicate *P* < 0.05

### Interaction between knowledge level and insulin therapy on glycemic control

To examine whether the effect of knowledge level on glycemic control varied by insulin use, an interaction model between knowledge level and insulin therapy was constructed (Table 4). The main effects analysis showed that, after adjusting for other factors, medium knowledge level (OR = 0.61, 95% CI: 0.45–0.83, *P* = 0.001) and insulin injection (OR = 0.59, 95% CI: 0.43–0.82, *P* = 0.002) were both negatively associated with good glycemic control, whereas the association between high knowledge level and glycemic control did not reach statistical significance (OR = 0.60, *P* = 0.066).

The interaction effect analysis revealed a significant positive interaction between high knowledge level and insulin therapy (interaction term OR = 4.60, 95% CI: 1.78–11.89, *P* = 0.002), while the interaction between medium knowledge level and insulin therapy was only marginally significant (interaction term OR = 1.67, 95% CI: 0.92–3.01, *P* = 0.090).

**Table 4:** Interaction analysis of knowledge level and insulin injection on glycemic control (*n* = 1495)

Variable	OR	SE	z value	*P* value	95% CI	
					Lower	Upper
Knowledge level						
Medium	0.61	0.09	-3.18	**0.001**	0.45	0.83
High	0.60	0.17	-1.84	0.066	0.35	1.03
Insulin injection	0.59	0.10	-3.13	**0.002**	0.43	0.82
Knowledge level × Insulin injection						
Medium	1.67	0.50	1.70	0.090	0.92	3.01
High	4.60	2.23	3.15	**0.002**	1.78	11.89

Note: Insulin injection is a binary variable (any insulin use, including combination therapy). Among the 1,495 participants, 320 (21.4%) were receiving insulin therapy. Within the insulin-treated group, 195 (61.0%) had low knowledge levels, 91 (28.3%) had moderate knowledge levels, and 34 (10.6%) had high knowledge levels. Among those not receiving insulin, the corresponding numbers were 889 (75.7%), 227 (19.3%), and 59 (5.0%), respectively. The model was adjusted for age, sex, duration of diabetes, current smoking, regular physical exercise, comorbid hypertension, and blood glucose monitoring adherence. Robust standard errors were used. Bold values indicate P<0.05

### Interaction between blood glucose monitoring adherence and insulin therapy on glycemic control

Using the same analytical strategy, an interaction model between blood glucose monitoring adherence and insulin therapy was constructed (Table 5). The main effects analysis showed that high blood glucose monitoring adherence was negatively associated with good glycemic control (OR = 0.67, 95% CI: 0.45–1.00, *P* = 0.049), whereas neither the main effect of medium adherence nor that of insulin injection reached statistical significance (*P* > 0.05).

The interaction effect analysis indicated no significant interaction between blood glucose monitoring adherence and insulin therapy (medium adherence × insulin: OR = 0.57, *P* = 0.263; high adherence × insulin: OR = 0.70, *P* = 0.407).

**Table 5:** Interaction analysis of blood glucose monitoring adherence and insulin injection on glycemic control (*n* = 1496)

Variable	OR	SE	z value	*P* value	95% CI	
					Lower	Upper
Blood glucose monitoring adherence						
Medium	0.86	0.20	-0.66	0.509	0.55	1.34
High	0.67	0.14	-1.96	**0.049**	0.45	1.00
Insulin injection	1.11	0.45	0.26	0.797	0.50	2.45
Blood glucose monitoring adherence × Insulin injection						
Medium	0.57	0.28	-1.12	0.263	0.22	1.52
High	0.70	0.30	-0.83	0.407	0.30	1.63

Note: Insulin injection is a binary variable. Among the 1,496 participants included in the interaction analysis after adjustment for all covariates, 1,086 (72.6%) had low, 317 (21.2%) moderate, and 93 (6.2%) high knowledge levels. By blood glucose monitoring adherence status, the distribution of knowledge levels was: good adherence (*n* = 1,008)—low 727 (72.1%), moderate 228 (22.6%), high 53 (5.3%); poor adherence (*n* = 338)—low 257 (76.0%), moderate 57 (16.9%), high 24 (7.1%); and moderate adherence (*n* = 150)—low 102 (68.0%), moderate 32 (21.3%), high 16 (10.7%). The model was adjusted for age, sex, duration of diabetes, current smoking, regular physical exercise, comorbid hypertension, and knowledge level. Robust standard errors were used. Bold values indicate P < 0.05

## Discussion and conclusion

Among patients with type 2 diabetes aged 40 years and older enrolled in the NEPHS in Mianyang City, the good glycemic control rate was 49.5%, which is basically consistent with the diabetes control rate in China in recent years [2], higher than the 7%–43.7% reported in Ethiopia, Moroccan, and other countries [11–13], and slightly lower than the HbA1c target achievement rate of 52.5% in the United States during 1988–2010 [14].

This study found that, under the premise of uniformly adopting HbA1c < 7% as the control standard, age ≥ 60 years was significantly positively correlated with good glycemic control. Although this result contradicts some studies [15], it is consistent with findings in Xinjiang, China [16]. The exact mechanism of this association remains unclear and may result from the combined action of multiple factors. First, the existence of survival bias should be considered: patients who have survived to age 60 and remain active in the community management cohort may themselves be relatively successful “survivors” in disease management; whereas patients with extremely poor glycemic control and severe complications may have dropped out or died earlier, leading to a relatively good control rate among the remaining elderly population. Second, the National Essential Public Health Service project designates the elderly, diabetes, and hypertension patients as priority management populations, providing them with higher-frequency follow-up and health monitoring. Such institutionalized intensive management may translate into better glycemic control. Regarding urban–rural distribution, the results of this study showed that rural residence was associated with higher odds of good glycemic control. This finding contradicts the results in rural areas of Xinjiang (where rural residence was a risk factor). This discrepancy suggests that the implementation effectiveness of the Essential Public Health Service may vary significantly across different regions. Meanwhile, the rural sample in this study may have been concentrated among people who have long followed medical advice, are relatively satisfied with their glycemic control, and are therefore willing to continue receiving community follow-up. In contrast, Xinjiang is vast with a sparse population, the radius of grassroots health services is too large, and the accessibility of diabetes management is limited, so rural residence constitutes an obstacle to glycemic control [16].

The results showed that regular physical exercise was a strong independent predictor of good glycemic control [17, 18], which is consistent with existing evidence that exercise improves insulin sensitivity. Exercise-induced improvements in skeletal muscle glucose uptake and reductions in visceral fat contribute importantly to enhanced insulin sensitivity and long-term glycemic management [19]. The higher odds of good glycemic control of comorbid hypertension on glycemic control appear contradictory, but in the context of increasingly prevalent multimorbidity, this finding may be explained by the fact that patients with multimorbidity receive closer medical monitoring and are more likely to adhere to comprehensive treatment regimens. This phenomenon is sometimes referred to as the “care cascade effect” in patients with multimorbidity, suggesting that the presence of hypertension may serve as a catalyst promoting overall disease management improvement [20], rather than exerting a direct biological protective effect.

Both oral hypoglycemic agent monotherapy and insulin monotherapy were associated with poor glycemic control, whereas combination therapy was only marginally significant. This finding more likely reflects disease severity rather than the ineffectiveness of the treatments themselves. The association between longer disease duration and poor glycemic control is biologically plausible and consistent with the natural history of type 2 diabetes. As the disease duration extends, progressive deterioration of islet β-cell function and gradual aggravation of insulin resistance [21, 22] make glycemic targets increasingly difficult to achieve [23]. Current smoking was a significant barrier to glycemic control, consistent with extensive literature documenting the adverse effects of tobacco use on insulin sensitivity and vascular health [24, 25]. Nicotine and other components in cigarette smoke can induce oxidative stress and endoplasmic reticulum stress, both of which exacerbate insulin resistance and impair islet β-cell function. The independent effect of smoking observed after multivariable adjustment highlights the importance of incorporating smoking cessation programs into routine diabetes management in community health services. Prior evidence indicates that obesity and poor glycemic control frequently co-occur [26]; however, the limited differences in glycemic control proportions across BMI categories observed in this study may have obscured the true relationship between obesity severity and glycemic control.

In this cross-sectional study, the relationship between knowledge level and glycemic control exhibited a complex nonlinear pattern, and this relationship was significantly influenced by insulin therapy. Univariate analysis showed that the poor control group actually had higher total knowledge scores, including basic knowledge, symptom knowledge, and management knowledge scores. However, this seemingly contradictory finding was substantially attenuated in the multivariable model: only medium knowledge level was significantly associated with poor glycemic control, while high knowledge level showed the same trend but did not reach statistical significance. This pattern suggests that knowledge alone, without effective translation into behavior, may be insufficient for glycemic management. Patients with higher knowledge levels may have greater awareness of disease complexity and thus report poorer perceived control; alternatively, higher knowledge may be a marker of heavier disease burden, as these patients may have engaged in more self-learning due to their disease needs. Interaction analysis provided critical clarification of this complex relationship. We found a significant positive interaction between high knowledge level and insulin therapy, indicating that knowledge significantly modified the detrimental association between insulin use and poor glycemic control. Specifically, among patients receiving insulin therapy, those with high knowledge levels achieved markedly better glycemic outcomes than those with lower knowledge. This finding carries important clinical implications: insulin therapy involves complex self-management skills, including dose adjustment, hypoglycemia recognition, and injection technique [27, 28]. When knowledge is insufficient, insulin therapy may paradoxically be associated with worse outcomes due to improper use or inadequate self-monitoring. This suggests that structured diabetes education should be intensified when initiating or intensifying insulin therapy. particularly in community settings where specialized diabetes education resources may be limited.

In contrast, no significant interaction was found between blood glucose monitoring adherence and insulin therapy. The main effect of self-rated high monitoring adherence was negatively associated with glycemic control, which may reflect reverse causation: patients with poor glycemic control may monitor more frequently precisely because of their poor status, driven by physician recommendations or personal concerns. However, regular HbA1c testing was positively associated with good glycemic control, suggesting that proactive monitoring by the medical system rather than patients’ self-measurement is the key mechanism promoting target achievement.

This study has several limitations. First, the cross-sectional design cannot establish causal relationships; the observed associations may be subject to reverse causation and unmeasured confounding. Second, The second-stage recruitment employed convenience sampling due to incomplete rosters and geographic inaccessibility in mountainous areas, which likely overrepresented actively engaged patients with better access to township health centers. To counteract the expected female predominance under this convenience sampling context—where women typically demonstrate higher compliance with community-based chronic disease management—a 1:1 sex quota was applied. While this quota ensured adequate male representation for stratified analyses, it deliberately distorted the true sex distribution of the managed population. Consequently, the overall glycemic control rate should be interpreted as characterizing “managed, accessible patients” rather than as a population prevalence estimate for all NEPHS-registered T2DM patients in Mianyang. Third, behavioral variables including regular exercise, smoking, alcohol consumption, and diet rely on patient recall, which may introduce information bias. Finally, caution is warranted when generalizing the findings to other healthcare systems or populations, as this study was restricted to patients enrolled in China’s National Essential Public Health Service.

In conclusion, this study identified multiple modifiable factors affecting glycemic control in patients with type 2 diabetes aged 40 years and older managed in the community in China, particularly regular physical exercise and smoking cessation. The significant interaction between diabetes knowledge and insulin therapy suggests the potential for a stratified intervention approach combining pharmacotherapy with primary self-management, though further validation is required prior to implementation. Future interventions should prioritize comprehensive strategies integrating systematic HbA1c monitoring, lifestyle intervention, and knowledge-enhanced insulin management to optimize glycemic outcomes in community settings.

## Supplementary Information

Below is the link to the electronic supplementary material.

Supplementary Material 1

## Data Availability

The data are not publicly available due to research ethical reasons and the corresponding author can provide further information on a reasonable request.
