# Supplementary material for: Characteristics and risk factors of glycemic control among middle-aged and elderly diabetes patients under management: a cross-sectional survey based on national essential public health services in Mianyang City
Source: BMC Endocr Disord. 2026 Aug 12;26:361. doi: 10.1186/s12902-026-02465-x (PMC13746399; doi:10.1186/s12902-026-02465-x)
Supplement: Supplementary file 1 [file 12902_2026_2465_MOESM1_ESM.docx]

**Questionnaire No.** □□□□□□□□

**Health Survey Questionnaire for Diabetes Patients Under Management in Mianyang City**

**Please fill in the blanks or tick "√" on the serial number of the corresponding option.**

**I. Basic Information**

**1.1 Age:** ______ years old

**1.2 Gender:** ① Male ② Female

**1.3 Height:** ______ cm

**1.4 Weight:** ______ kg (Kg)

**1.5 Waist Circumference:** ______ cm

**1.6 Blood Pressure:** ______ mmHg (Format: Systolic-Diastolic)

**1.7 Your current or pre-retirement occupation:**

① Civil servant ② Teacher ③ Medical staff ④ Other public institution staff

⑤ Student ⑥ Farmer ⑦ Worker ⑧ Other enterprise staff

⑨ Other ______

**1.8 Education Level:**

① Illiterate or barely literate ② Primary school ③ Junior high school

④ Senior high school / Vocational high school / Technical secondary school ⑤ College / Bachelor's degree ⑥ Master's degree or above

**1.9 Marital Status:**

① Unmarried ② Married ③ Divorced / Widowed

**1.10 Your annual household income:**

① <10,000 RMB ② 10,000-30,000 RMB (including 30,000) ③ 30,000-50,000 RMB (including 50,000)

④ 50,000-100,000 RMB ⑤ >100,000 RMB

**1.11 Current number of people living together:** ______ person(s)

**II. Disease Information (Please tick "√" on the serial number of the corresponding option.)**

**2.1 Your age at diagnosis of diabetes:** ______ years old.

**2.2 Your average monthly medical expenses for diabetes treatment:** ______ RMB

**Of which, the out-of-pocket expenses are:** ______ RMB

**2.3 Do any of your family members (parents or siblings) have diabetes?**

① Yes ② No ③ Don't know

**2.4 Have you ever been diagnosed with any of the following diseases? (Multiple choices allowed)**

① Hypertension ② Coronary heart disease ③ Hyperlipidemia ④ Stroke

⑤ Diabetic foot ⑥ Diabetic nephropathy ⑦ Retinopathy ⑧ Neuropathy

⑨ None ⑩ Other

**2.5 How did you discover that you had diabetes?**

① Actively tested due to concern about diabetes

② Sought medical attention due to physical discomfort

③ Routine physical examination

④ Discovered during treatment for other diseases

⑤ None of the above

**If you chose ② in Question 2.5, please continue to answer "2.6"; if you chose any of ①, ③, ④, or ⑤, please skip to "3.1".**

**2.6 What physical discomforts did you experience at that time? (Multiple choices allowed)**

① Polyphagia (eating more) ② Polydipsia (drinking more) ③ Polyuria (urinating more) ④ Significant weight loss in a short period

⑤ Skin itching ⑥ Recurrent urinary tract infections ⑦ Wounds not healing easily ⑧ Other

**III. Knowledge**

**3.1 How would you rate your understanding of diabetes?**

① Very well informed ② Relatively well informed ③ Little knowledge ④ No knowledge at all

**3.2 Do you think diabetes can be cured?**

① Can be cured **② Cannot be cured** ③ Don't know

**3.3 The ideal fasting blood glucose control target for Type 2 diabetes patients is:**

**① 3.9-7.2 mmol/L** ② 8.0-10.0 mmol/L ③ Don't know

**3.4 The ideal 2-hour postprandial blood glucose control target for Type 2 diabetes patients is:**

**① <10.0 mmol/L** ② <12.0 mmol/L ③ Don't know

**3.5 The ideal glycated hemoglobin (HbA1c) control target for Type 2 diabetes patients is:**

**① 5.0%-7.0%** ② 7.0%-9.0% ③ Don't know

**3.6 Manifestations of hypoglycemia: (Multiple choices allowed)**

**① Dizziness ② Pale complexion ③ Palpitations ④ Cold sweats**

**⑤ Extreme hunger ⑥ General fatigue** ⑦ Don't know

**3.7 Manifestations of hyperglycemia: (Multiple choices allowed)**

**① Thirst ② Drinking more ③ Urinating more ④ Feeling tired**

**⑤ Blurred vision** ⑥ Don't know

**3.8 The significance of blood glucose monitoring lies in: (Multiple choices allowed)**

**① Helping adjust dietary plan ② Helping adjust medication**

**③ Helping adjust exercise mode and intensity ④ Monitoring disease progression** ⑤ Don't know

**3.9 Common complications of diabetes include: (Multiple choices allowed)**

**① Diabetic nephropathy ② Retinopathy ③ Diabetic foot**

**④ Neuropathy ⑤ Cardiovascular disease** ⑥ Don't know

**3.10 Comprehensive treatment of diabetes includes: (Multiple choices allowed)**

**① Psychological therapy ② Dietary therapy ③ Exercise therapy ④ Drug therapy ⑤ Health education** ⑥ Don't know

**3.11 High-risk groups for diabetes include: (Multiple choices allowed)**

**① Age ≥45 years ② Hypertensive patients ③ Obese individuals ④ People with family history**

**⑤ Women with history of macrosomia (birth weight ≥4 kg)**

**⑥ People with predominantly sedentary lifestyle** ⑦ Don't know

**3.12 Regarding blood glucose control, which of the following statements are INCORRECT? (Multiple choices allowed)**

**① The lower the blood glucose control, the better.**

**② For diabetes monitoring, testing blood glucose alone is sufficient.**

**③ Once blood glucose is controlled within the normal range, there is no need for blood glucose monitoring and drug therapy.**

④ Don't know

**3.13 Regarding diabetes, which of the following statements are INCORRECT? (Multiple choices allowed)**

**① Diabetes is mainly caused by eating too much sugar or sweets.**

**② Once diagnosed with diabetes, taking medication alone is sufficient.**

**③ Although diagnosed with diabetes, if there is no discomfort, there is no need for examination or treatment.**

④ Don't know

**3.14 Do you know which of the following conditions are NOT suitable for exercise? (Multiple choices allowed)**

**① Poor blood glucose control ② Severe complications ③ Infection**

④ No restrictions ⑤ Don't know

**3.15 Regarding diabetes diet, which of the following statements are INCORRECT? (Multiple choices allowed)**

**① Some health supplements can cure diabetes.**

**② Dietary therapy for diabetes mainly refers to controlling sugar or staple food intake.**

**③ As long as you take more hypoglycemic drugs, you can skip dietary control.**

④ Don't know

**3.16 What should diabetic patients pay attention to during exercise? (Multiple choices allowed)**

**① Choose appropriate exercise mode according to physical condition ② Exercise for about half an hour every day**

**③ Start exercising one hour after meals ④ Exercise intensity should not be excessive**

**⑤ Carry candy to avoid hypoglycemia** ⑥ Don't know

**3.17 Regarding the diet of diabetic patients, which of the following do you think are correct? (Multiple choices allowed)**

**① Three meals at regular times and in fixed amounts, limit starch intake**

**② Prefer light, low-salt, and low-fat diet**

③ Melon seeds and peanuts and other nuts can be eaten without limit

**④ Eat more whole grains and vegetables**

**⑤ Under the condition of controlling total calorie intake, food variety can be diversified**

**IV. Lifestyle and Behavior**

**4.1 What measures do you take to control blood glucose? (Multiple choices allowed)**

① Oral hypoglycemic drugs ② Insulin injection ③ Dietary control

④ Exercise ⑤ No measures taken

**4.2 How often do you usually measure glycated hemoglobin (HbA1c)?**

① Never measure ② Once a year ③ Once every six months ④ Once every three months ⑤ Once a month

**4.3 Do you measure blood glucose regularly as required by your doctor?**

① Can measure as required by the doctor

② Measure as required by the doctor in most cases

③ Decide whether to measure blood glucose based on personal feelings

**4.4 How many times do you exercise per week?** (Those engaged in moderate-to-heavy physical labor are considered to exercise regularly)

① Never exercise ② Occasionally ③ Regularly (≥3 times/week and ≥30 minutes/time) ④ Irregularly

**4.5 What types of physical exercise do you do? (Multiple choices allowed)**

① Aerobic exercise such as brisk walking, jogging ② Tai Chi or Baduanjin, etc. ③ Resistance exercise (elastic bands, dumbbells, etc.)

④ Racket sports such as badminton, table tennis, etc. ⑤ Stretching exercise: yoga, Pilates, etc.

**4.6 Your dietary habits (Multiple choices allowed):** ① Prefer salty taste ② Prefer oily taste

③ Often eat vegetables (≥1 time/day) ④ Rarely eat vegetables ⑤ Often eat fruits (≥3 times/week) ⑥ Rarely eat fruits

⑦ Milk or yogurt (≥250 ml/day) ⑧ Don't drink or rarely drink milk

**4.7 What time do you usually have breakfast?**

① 6:00-7:00 ② 7:01-8:00 ③ 8:01-9:00

④ After 9:00 ⑤ Skip breakfast

**4.8 Your medication status is:**

① Take medication as required by the doctor

② Take medication as required by the doctor in most cases

③ Stop medication when condition is stable

④ Increase or decrease medication on your own based on blood glucose monitoring results

⑤ Other

**4.9 Do you currently smoke?**

① Smoke ② Used to smoke, but not now ③ Do not smoke

**4.10 Do you currently drink alcohol?**

① Drink often ② Drink occasionally ③ Do not drink

**V. Diabetes Information Acquisition and Processing**

**5.1 What channels do you usually use to learn about diabetes information? (Multiple choices allowed, no more than 5)**

① Doctor ② Family members ③ Friends or colleagues ④ Newspapers, books ⑤ Radio

⑥ Television ⑦ Internet ⑧ Mobile text messages ⑨ Other

**5.2 Do you trust the diabetes information from the following sources? (Please tick "√" in the corresponding grid)**

| Information Source | ① Trust | ② Relatively trust | ③ Do not trust | ④ Have not used this source, cannot judge |
| --- | --- | --- | --- | --- |
| 1 Doctor |  |  |  |  |
| 2 Family members |  |  |  |  |
| 3 Friends or colleagues |  |  |  |  |
| 4 Newspapers, books |  |  |  |  |
| 5 Radio |  |  |  |  |
| 6 Television |  |  |  |  |
| 7 Internet |  |  |  |  |
| 8 Mobile text messages |  |  |  |  |

**5.3 Do you think your diabetes knowledge can meet your own needs?**

① Already sufficient ② Need some more ③ Lack a lot ④ Urgently need to learn

**5.4 In the past 12 months, have you used the Internet? (Please choose one)**

① Yes ② No *(Survey ends, thank you for your cooperation!)*

**5.5 In the past 12 months, have you often used the Internet to search for health or disease information?**

① Often ② Sometimes ③ Rarely ④ Never

**5.6 Do you think the health or disease information on the Internet is useful?**

① Very useful ② Relatively useful ③ Not very useful ④ Completely useless

******Survey ends, thank you for your cooperation!******
